# Chemoprevention of hepatocellular carcinoma using N-acetylgalactosamine-conjugated siRNAs

**DOI:** 10.1242/dmm.052370

**Published:** 2025-09-01

**Authors:** Gianna Maggiore, Meng-Hsiung Hsieh, Amaey Bellary, Purva Gopal, Lin Li, Jason Guo, David Hsiehchen, Tulin Dadali, Wendy Broom, Martin Maier, Hao Zhu

**Affiliations:** ^1^ Children's Research Institute, Departments of Pediatrics and Internal Medicine, Center for Regenerative Science and Medicine, Simmons Comprehensive Cancer Center, Children's Research Institute Mouse Genome Engineering Core, University of Texas Southwestern Medical Center, Dallas, TX 75390, USA; ^2^ Department of Pathology, University of Texas Southwestern Medical Center, Dallas, TX 75390, USA; ^3^ Department of Internal Medicine, Division of Hematology-Oncology, Simmons Comprehensive Cancer Center, University of Texas Southwestern Medical Center, Dallas, TX 75390, USA; ^4^ Alnylam Pharmaceuticals, Cambridge, MA 02142, USA

**Keywords:** Cancer prevention, Hepatocellular carcinoma (HCC), GalNAc-siRNA, Metabolic dysfunction-associated steatohepatitis (MASH)

## Abstract

The ability to prevent hepatocellular carcinoma (HCC) in patients with chronic liver disease remains an unmet clinical need. We performed a head-to-head comparison of N-acetylgalactosamine (GalNAc)-conjugated small interfering RNA (siRNA)-mediated inhibition of five genes (*CDK1*, *PD-L1*, *CTNNB1*, *SMYD3*, *ANLN*) to prevent cancer in four distinct autochthonous HCC mouse models. siRNA targeting *Cdk1* and *Anln* (siCdk1 and siAnln, respectively) increased overall survival in the *CTNNB1/MYC* hydrodynamic transfection (HDT) model, in which HCC formation is driven by oncogenes. Both long-term and transient dosing of siCtnnb1 or siAnln prevented cancer development in the *NRAS^G12V^*/*shp53*-driven HDT model*.* siCdk1 and siAnln prevented cancer in a diethylnitrosamine/phenobarbital model, in which tumor formation is driven by mutagenesis and chemical injury. Moreover, siCtnnb1 and siAnln decreased cancer development in a metabolic dysfunction-associated steatohepatitis (MASH) model driven by a Western diet and carbon tetrachloride (CCl_4_). Given that the use of siAnln was effective in several models, we validated *Anln* effects using Cre-lox and found that histologic features of MASH and HCC development were independently reduced. This demonstrates that siRNAs are safe and effective in preventing HCC in a large panel of preclinical cancer models, and identifies ANLN as an effective chemoprevention target.

## Teaser

**Editor's choice:** Comparison between five N-acetylgalactosamine-conjugated siRNAs in several mouse models of liver cancer found *ANLN* to be a promising target for hepatocellular carcinoma chemoprevention.

## INTRODUCTION

Liver cancer is the fourth leading cause of cancer-related death in the world, with hepatocellular carcinoma (HCC) accounting for the majority of cases (Sung et al., 2021). Chronic liver damage eventually leads to liver cirrhosis, and 2-3% of patients with cirrhosis progress to HCC each year (Reddy et al., 2023). Regardless of etiology, HCC has a 5-year survival rate of <20% (Brar et al., 2020). Prevention in patients with cirrhosis would be the most effective approach for reducing HCC burden and mortality, especially given that we are able to identify patients who are at increased risk of developing HCC (Fujiwara et al., 2021). However, the criteria for successful prevention, which include a high level of safety and effectiveness, have not yet been met with the approaches explored to date. Agents, such as aspirin and statins, have shown some promise, as has etiology-specific treatments such as antiviral therapies for hepatitis B (HBV) and C (HCV), but no randomized control trials have been carried out to definitively demonstrate cancer prevention (Rocha et al., 2023; Suzuki et al., 2023). Even in the setting of curative therapies for HCV or HBV, elimination of the virus reduces but does not eliminate HCC risk (Rocha et al., 2023; Yang et al., 2022). Furthermore, obesity/diabetes can lead to metabolic dysfunction-associated steatotic liver disease (MASLD) with metabolic dysfunction-associated steatohepatitis (MASH) now being the most common liver disease (Younossi et al., 2023), and the first treatment has only recently been approved (Harrison et al., 2024). Given the lethality of HCC and an identifiable high-risk population, chemoprevention is both an unmet need and an enormous clinical opportunity to reduce cancer deaths (Suzuki et al., 2023).

A major knowledge gap is that there is no consensus about optimal targets for liver cancer chemoprevention. Furthermore, there have been no head-to-head comparisons of promising approaches for HCC chemoprevention. We have previously assessed the potential of therapeutically induced inhibition of cytokinesis and polyploidization in hepatocytes, physiologic events that naturally protect the liver from cancer (Lin et al., 2020; Zhang et al., 2018a,b). Continuous or transient knockdown of the actin-binding protein anillin (ANLN) prevented the formation of liver tumors (Lin et al., 2020; Zhang et al., 2018a,b). Remarkably, inhibition of ANLN does not disrupt liver function or healthy regeneration in preclinical models, even when confronted with the kind of chronic liver injury seen in cirrhosis patients (Lin et al., 2020). While ANLN could be exploited for HCC chemoprevention, it is possible that other gene targets with strong rationales as anti-cancer agents have more potent or less toxic effects. To address this hypothesis, four other targets, in addition to ANLN, were nominated based on previous data demonstrating that genetic deletion or pharmacological inhibition can inhibit cancer development.

Preclinical studies in mice, in addition to genetic data obtained from human studies, highlight the role these gene targets play in HCC development. Cyclin-dependent kinase 1 (CDK1) is overexpressed in human liver cancer and liver-specific knockout (KO) of *Cdk1* in mice prevents liver cancer (Diril et al., 2012). CDK1 is a central driver of mitosis and, like deletion of *Anln*, absence of CDK1 does not impair function or regeneration after liver injury (Diril et al., 2012). Liver-specific KO of *Cdk1* has been shown to almost completely prevent HCC tumorigenesis driven by RAS activation and *Tp53* silencing (Diril et al., 2012). Programmed death ligand 1 (PD-L1, officially known as CD274) is an immune checkpoint molecule that suppresses the immune response, and anti-PD-L1 antibodies are a mainstay of modern cancer treatment (Bellmunt et al., 2017; Brahmer et al., 2015; Motzer et al., 2018; Robert et al., 2015). Inhibition of PD-L1 prevents liver cancer in a diethylnitrosamine (DEN) and carbon tetrachloride (CCl_4_) mouse model (Chung et al., 2020). β-catenin (CTNNB1), a key component of the Wnt/β-catenin signaling pathway, is a commonly mutated oncogene in liver cancer. While mice with liver KO of *Ctnnb1* exhibit impaired tissue regeneration after injury (Tan et al., 2006), knockdown via lipid-nanoparticles (LNPs) by using small interfering RNA (siRNA) reduces HCC in mutant β-catenin-driven mouse models (Tao et al., 2017). SET and MYND-containing domain 3 (SMYD3), a protein methyltransferase with multiple histone and non-histone targets, is overexpressed in human HCC, and deletion of *Smyd3* prevents DEN-induced liver tumor development in mice (Sarris et al., 2016; Zhang et al., 2021). Here, our objective was to evaluate these target genes/proteins in a head-to-head fashion to identify the most effective candidates for HCC prevention.

To avoid extra-hepatic toxicity, HCC chemoprevention therapy is best delivered in a liver-specific fashion. So far, this has not been attempted clinically, in part because the optimal gene target is unknown. Hepato-tropic siRNA conjugates are approved by the US Food and Drug Administration (FDA) and poised for use as HCC preventive agents (Springer and Dowdy, 2018). Over the last two decades, the dual challenge of delivering siRNAs and inducing a robust gene knockdown in non-transformed hepatocytes have been met (Nair et al., 2014). Since 2018, the FDA has approved six hepatocyte-targeted siRNA therapies; i.e. (i) patisiran for amyloidosis, (ii) givosiran for acute hepatic porphyria, (iii) lumasiran for primary hyperoxaluria type 1, (iv) inclisiran for heterozygous familial hypercholesterolemia or clinical atherosclerotic cardiovascular disease, (i) vutrisiran for ATTR amyloidosis and (vi) nedosiran for primary hyperoxaluria (Adams et al., 2018; Balwani et al., 2020; Baum et al., 2023; Garrelfs et al., 2021; Habtemariam et al., 2021; Ray et al., 2020). Alnylam Pharmaceuticals has shown that a single dose of siRNA targeting proprotein convertase subtilisin/kexin type 9 (PCSK9) can result in significantly reduced protein levels 180 days later (Ray et al., 2017; Ray et al., 2020), suggesting that a potential chemopreventive approach can be conveniently paired with standard surveillance schedules. In contrast to small-molecule inhibitors that can cause multi-organ on-target toxicity, the hepato-tropic nature of these siRNA conjugates is a logistical advantage because there are virtually no on- or off-target effects outside the liver (Springer and Dowdy, 2018). The current standard GalNAc-conjugated siRNA delivery strategy only leads to hepatocyte uptake of siRNAs (Springer and Dowdy, 2018). This siRNA delivery platform can be applied to genetic targets in liver disease, including HCC prevention. Thus, GalNAc-siRNA represents an innovative, clinically established drug-delivery approach regarding chemoprevention of HCC.

To test these siRNA-based approaches, we conducted a rigorous comparative analysis of the above target genes using a diverse panel of HCC mouse models. Multiple autochthonous mouse models of liver cancer are rarely used to even test single gene targets, much less in the context of a head-to-head comparison of five targets. We first tested two aggressive models to study cancer formation in the context of oncogenic drivers by using *CTNNB1/MYC* and *NRAS^G12V^*/*shp53* mouse models of HCC. We used a chemical model by combining mutagenesis caused by early exposure to DEN and forced proliferation due to low but chronic doses of phenobarbital (PB). Finally, we used a long-term mouse model combining a Western diet (WD) [20-23% fat by weight (40-45% kcal from fat), milkfat (saturated fatty acids >60% of total fatty acids), 1.25% cholesterol] with high-sugar water and chronic, low doses of CCl_4_ to model human MASH and subsequent progression to HCC. We believe that five gene targets tested in four independent mouse models of HCC – i.e. *CTNNB1/MYC*, *NRAS^G12V^/shp53*, DEN/PB, and WD and CCL4 – will provide the greatest chance of yielding an optimal chemoprevention strategy.

## RESULTS

### Testing siRNA chemoprevention in the CTNNB1/MYC HCC model

We first tested siRNA chemoprevention in a hydrodynamic transfection (HDT)-induced HCC model, which allowed us to examine cancer development in the context of oncogenic mutations. Mice were hydrodynamically injected through the tail vein with a large volume of saline-containing plasmids of interest (Chen and Calvisi, 2014). The large volume leads to cardiac congestion and retrograde flow to the liver, where the plasmids are taken up by hepatocytes. This model facilitates rapid tumor development and allows the flexible testing of different cancer drivers or therapeutic targets.

**Fig. 1.**
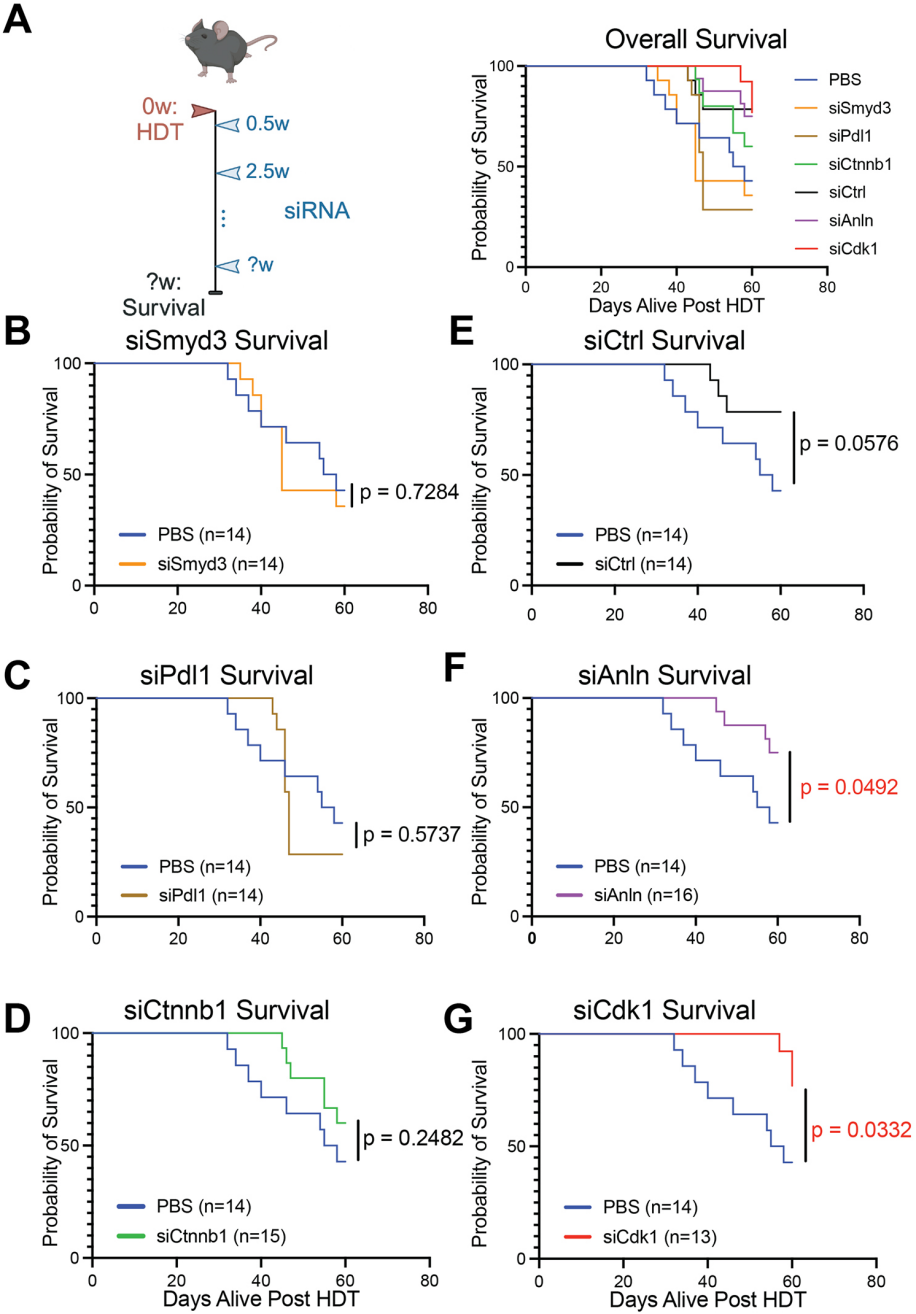
**siCDK1 and siANLN extended survival in the *CTNNB1/MYC* HCC model.** (A) Schematic of the *CTNNB1/MYC* mouse model. Male C57BL/6J mice underwent HDT injection at 8 weeks of age, i.e. start timepoint of 0 weeks (0w). siRNA injections started 4 days later (0.5w) and occurred every other week at a dose of 5 mg/kg (2.5w to ?w). Mice were followed for survival. To the right is a Kaplan–Meier curve with all groups. (B-G) Kaplan-Meir curves for survival of *CTNNB1/MYC* mice injected with siSmyd3 (B), siPdl1 (C), siCtnnb1 (D), siCtrl (E), siAnln (F) and siCDk1 (G). Only siCdk1 and siAnln resulted in significantly increased survival compared to PBS controls.

Male mice were injected via the tail vein with a combination of *myc-PT3EF1a* (Liu et al., 2017) and *pT3-EF1aH N90-CTNNB1* (Tward et al., 2007) transposon plasmids in addition to a Sleeping Beauty Transposase (hereafter referred to as SB100) (Tward et al., 2007) to induce integration into hepatocyte genomes. Three days after HDT, subcutaneous siRNA injections were initiated and given every 14 days at a dose of 5 mg/kg of mouse (Fig. 1A). We administered siRNA 3 days after HDT to allow enough time for transposon integration but before tumor initiation. This delay also allowed animals to recover from HDT-induced liver and cardiac stress. Initiating siRNA after integration but before tumor formation ensured observed effects reflected tumor prevention rather than treatment. The 14-day dosing schedule was determined by Alnylam Pharmaceuticals and based on previous data (Foster et al., 2018). Seven groups of mice were created: in five groups, each mouse was injected siRNA targeting one specific gene per group, i.e. *Cdk1*, *Pdl1*, *Ctnnb1*, *Anln* or *Smyd3* (hereafter referred to as siCdk1, siPdl1, siCtnnb1, siAnln or siSmyd3, respectively); in the sixth group, each mouse was injected with PBS-based control; and in the seventh group, each mouse was injected with a siRNA targeting luciferase (siCtrl). Mice were followed for survival and sacrificed when their body condition score was 1 (out of 5).

Around 35 days after HDT, we began to observe bulging flanks, a sign of extensive liver tumor development. For the PBS control group, the median overall survival was 56.5 days. For the siSmyd3 group, the median overall survival was 45 days (Fig. 1B, *P*=0.7284). For the siPdl1 group, the median overall survival was 47 days (Fig. 1C, *P*=0.5737). The siCtnnb1, siCtrl, siCdk1 and siAnln groups did not reach the median overall survival within 60 days of HDT, indicating increases in survival. While siCtnnb1-treated mice had increased survival, this was not significant (Fig. 1D, *P*=0.2482). Mice treated with siCtrl also had increased survival compared to the PBS mice, and although this increase did not reach significance, this led us to hypothesize that siCtrl exerted off-target effects (Fig. 1E, *P*=0.0576). Mice treated with siAnln or siCdk1 had significant increases in survival (Fig. 1F,G, *P*=0.0492 and *P*=0.0332, respectively). Protein knockdown was evaluated in livers with a mixture of malignant and non-malignant cells (Fig. S1A). SMYD3, PD-L1, CTNNB1 or CDK1 protein levels were reduced by 28%, 26%, 80% or 40%, respectively. Surprisingly, ANLN protein levels increased, suggesting compensatory transcriptional upregulation of *ANLN* after an initial period of knockdown. We confirmed that siAnln was able to suppress *ANLN* levels in healthy livers 3 days after siAnln delivery (Fig. S1B). Altogether, several of the candidate targets were effective in an aggressive *CTNNB1/MYC*-driven HCC model.

### Testing siRNA chemoprevention in the NRAS^G12V^/shp53 HCC model

**Fig. 2.**
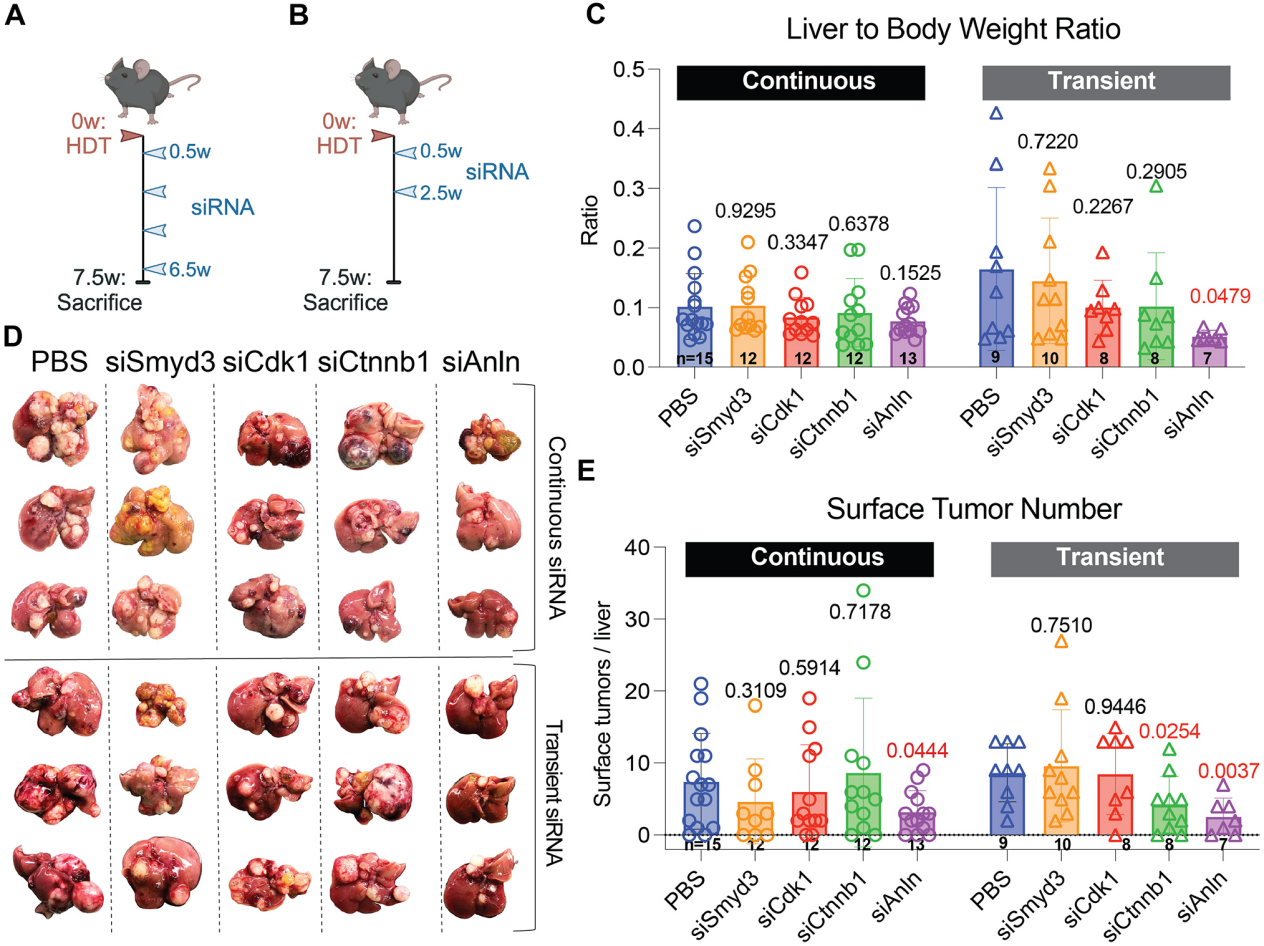
**siCtnnb1 and siAnln decreased tumor burden in the *NRAS^G12V^*/*shp53* HCC model.** (A) Schematic of *NRAS^G12V^*/*shp53* mouse model receiving continuous siRNA injections. Male C57BL/6J mice underwent HDT injection at 8 weeks of age, i.e. start timepoint of 0 weeks (0w). Injections of siRNA (5 mg/kg) started 4 days later (0.5w) and continued every other week (2.5w, 4.5w, 6.5w). Mice were sacrificed 1 week after the fourth siRNA dose (7.5w). (B) Schematic of *NRAS^G12V^*/*shp53* mouse model receiving transient siRNA injections. Male C57BL/6J mice underwent HDT injection at 8 weeks of age (0w). Injections of siRNA (5 mg/kg) started 4 days later and continued every other week for a total of two doses (0.5w and 2.5w). Mice were sacrificed 5 weeks after the second siRNA dose (7.5w). (C) Liver-to-body-weight ratios for *NRAS^G12V^*/*shp53* mice after continuous (circles) and transient (triangles) siRNA treatment. (D) Gross liver images from *NRAS^G12V^*/*shp53* mice after continuous (top three rows) and transient (bottom three rows) treatment with siRNA. (E) Quantification of surface tumors for *NRAS^G12V^*/*shp53* mice after continuous (circles) and transient (triangles) siRNA treatment. For panels C and E, the *P*-values with respect to the PBS group are shown above each group.

Because siCtnnb1 targets one of the oncogenes in the *CTNNB1/MYC* model, we next tested our siRNAs in an HCC model driven by oncogenic driver genes unrelated to one of the GalNAc-siRNA targets. We tested another aggressive HCC model driven by *NRAS^G12V^* and *shp53* (Wiesner et al., 2009). Similar to the previous model, male mice were injected with the transposons and transposase SB100. Three days after HDT, subcutaneous siRNA injections were initiated and were given every 14 days at a dose of 5 mg/kg of mouse (Fig. 2A). Owing to its potential off-target effects, we removed siCtrl from our subsequent investigations; we also omitted further use of siPdl1 and siSmyd3 because they performed poorly in the *CTNNB1/MYC* model. In the *NRAS^G12V^*/*shp53* model, we elected to use the number of tumors as the endpoint of the study, given the longer survival of this HCC model. *NRAS^G12V^*/*shp53* model mice were sacrificed 1 week after the fourth siRNA dose and livers were examined for tumor burden. There was no difference in liver-to-body-weight ratios between any of the siRNA groups, although the siCdk1 and siAnln weights tended to be lower (Fig. 2C, open circles). When surface tumor numbers were quantified, only siAnln-treated mice had significant reductions, with an average of 3.2±3 tumors/liver compared to 7.5±6.7 tumors/liver in the PBS group (mean±s.d.; Fig. 2D,E, open circles). While none of the other groups had significant differences in surface tumor numbers, siCdk1-treated mice had 6.1±6.4 tumors/liver and siSmyd3-treated mice had 4.7±5.9 tumors/liver. siCtnnb1-treated mice had a trend towards more tumors, with 8.7±10.3 tumors/liver.

It would be ideal if transient siRNA dosing could achieve long-term cancer prevention, since fewer doses might limit long-term toxicities. Previous work has also shown that transient ANLN knockdown is sufficient to prevent HCC development through increased hepatocyte polyploidization (Zhang et al., 2018b). In addition to continuous siRNA dosing, we determined if transient knockdown of target genes can alter tumor burden in the *NRAS^G12V^*/*shp53* model. Mice were initiated on siRNA treatment 3 days after HDT, but only two siRNA injections in total were administered (Fig. 2B). Mice were sacrificed at the same time as the cohort that continuously received siRNA doses. In the continuously treated cohort, we found no differences between siRNA groups in terms of the liver weight to body weight ratio (Fig. 2B, open circle). However, mice given only two doses of siAnln had lower liver-to-body-weight ratios than mice from the other groups (Fig. 2B, open triangles). The number of surface tumors was also decreased in the siAnln-injected group (mean of 2.5±2.5 tumors/liver) and siCtnnb1-injected group (mean of 4±3.9 tumors/liver) compared to that of the PBS group (8.6±4.0 tumors/liver) (Fig. 2D,E, open triangles). Thus, both continuous and transient siAnln was sufficient to prevent *NRAS^G12V^*/*shp53*-induced liver cancer. However, only transient, but not continuous, siCtnnb1 was sufficient to prevent *NRAS^G12V^*/*shp53*-induced liver cancer. siCdk1-treated mice had similar a number of tumors compared to that of the PBS group, averaging 8.6±5.6 tumors/liver. siSmyd3-treated mice showed a trend towards increased tumor numbers, averaging 10.5±7.8 tumors/liver.

Western blots were used to quantify knockdown of proteins after targeting their genes by siRNAs. In the continuously treated mice, SMYD3, CTNNB1, CDK1 and ANLN protein levels were reduced by 37%, 29%, 22% and 7%, respectively (Fig. S2A). Only the changes in SMYD3 levels were significant. In transiently treated mice, only CTNNB1 was significantly reduced (by 14%), while levels of other target proteins were unchanged or slightly increased (Fig. S2B). This was expected, given the 5-week timeframe between the last dose of siRNA and the time of harvest. A critical question about GalNAc-siRNAs is whether or not they can enter and successfully knockdown cancers after they are established. To answer this, we assessed target protein knockdown in harvested tumors from continuously treated *NRAS^G12V^*/*shp53* mice. We found variable knockdown efficiency, with some tumors exhibiting substantial reduction in target protein levels while others showed modest changes (Fig. S2C). These findings indicated that successful knockdown of target genes in HCCs is possible but that it is highly dependent on the potency of the siRNA used. Even without genetic knockdown within cancers, GalNAc-siRNA can prevent HCC due to gene knockdown in hepatocytes.

### Testing siRNA chemoprevention in the DEN/PB HCC model

**Fig. 3.**
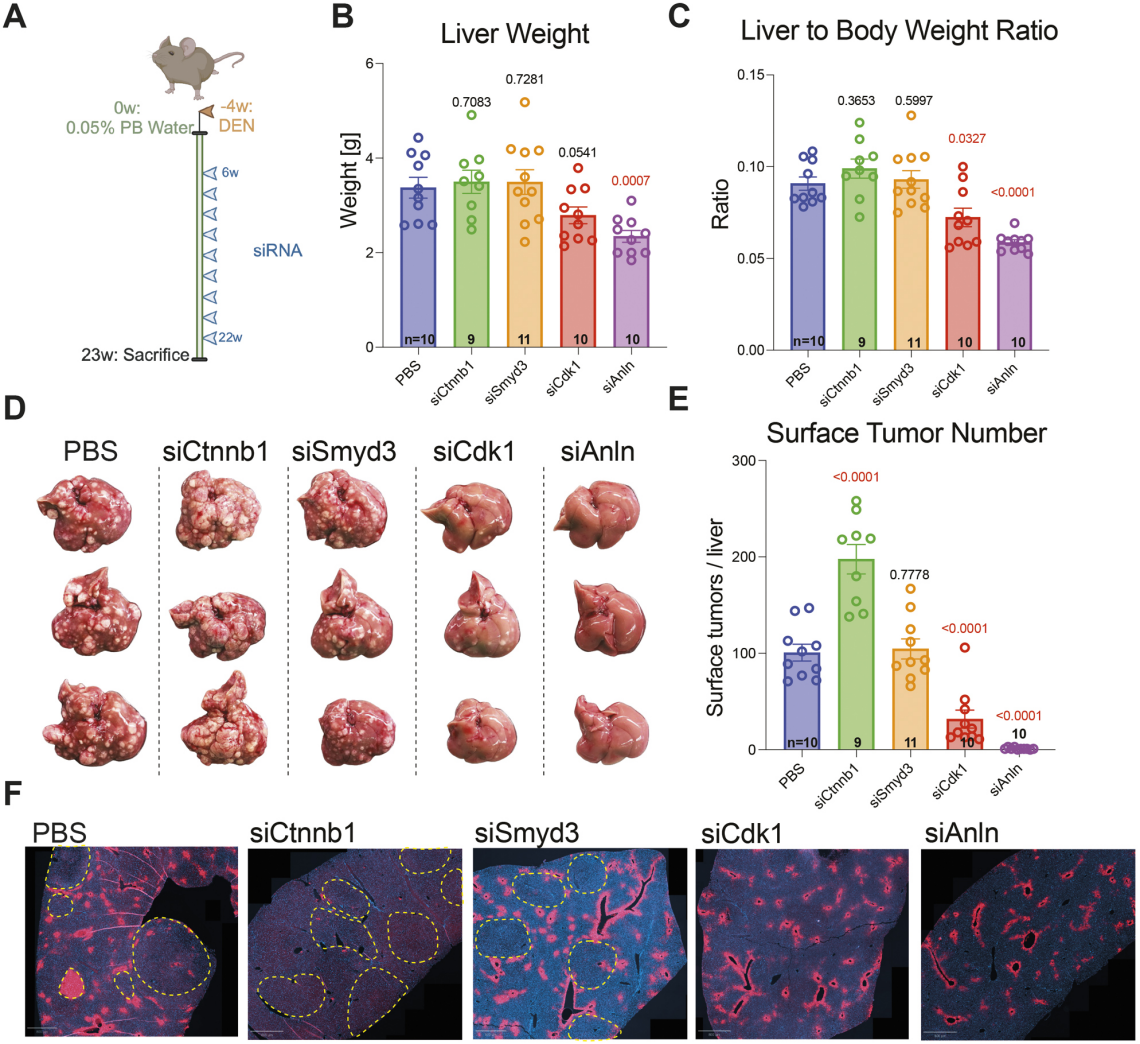
**siCdk1 and siAnln decreased tumor burden in the DEN/PB HCC model.** (A) Schematic overview of DEN/PB mouse model. At 2 weeks of age, male mice on the C3H strain background were given a single injection of DEN (25 mg/kg) 4 weeks before the start timepoint (-4w). At 6 weeks of age, mice were placed on 0.05% PB drinking water [start timepoint of 0 weeks (0w)]. Mice were injected with siRNA every other week starting at 12 weeks of age (6w), for a total of nine doses (with 5 mg/kg per dose) and sacrificed 1 week after the final dose (23w). (B) Liver weights for DEN/PB mice at 23 weeks after starting PB water, i.e. the final time point. (C) Liver-to-body-weight ratio for DEN/PB mice at the final time point. (D) Representative images of livers from DEN/PB mice at the final time point. (E) Surface tumor numbers were quantified using front and back images of each liver. (F) Representative immunofluorescence images of DEN/PB livers obtained from mice as indicated. Staining was against GS (red) to indicate the degree of WNT activation. Tumors are outlined by yellow dashed lines. One GS+ tumor was seen in the PBS group. Nuclei were stained with DAPI (blue). Scale bars: 800 μm. For panels B, C and E, the *P*-values with respect to the PBS group are shown above each group.

We next tested siRNA chemoprevention in a mouse cancer model driven by a single high dose of diethylnitrosamine (DEN) followed by ongoing dosing of phenobarbital (PB). DEN acts as the initiating carcinogenic agent, while PB acts as a tumor-promoting agent (Heindryckx et al., 2009; Klaunig et al., 1988). In this HCC model, mice were injected once with DEN at 2 weeks of age followed by access to drinking water supplemented with 0.05% PB at 6 weeks of age. At 12 weeks of age, subcutaneous injections of siRNA (5 mg/kg) were initiated and given every 14 days. After 23 weeks and nine doses of siRNA mice were sacrificed (Fig. 3A). No mice showed outward signs of illness or died prior to the endpoint of the study. While there was no difference in body weight between groups at study endpoint, siCdk1-treated mice trended towards liver weight decrease and siAnln-treated mice had significantly decreased liver weights (Fig. 3B). siCdk1- or siAnln-treated mice also had a significantly decreased liver-to-body-weight ratio (Fig. 3C). Both siCdk1- or siAnln-treated mice groups also had significantly reduced tumor burden (31.8±29.4 or 1.2±1.0 tumors/liver, respectively; mean±s.d.) compared to that of the PBS group (100.7±27.7 tumors/liver) (Fig. 3D,E). Remarkably, injection of siAnln resulted in a 84-fold reduction in HCC. siCtnnb1-treated mice had a significantly (*P*<0.0001) increased tumor burden, with an average of 197.67±45.7 tumors/liver (Fig. 3D,E).

This DEN/PB model was previously shown to give rise to tumors due to β-catenin mutations (Loeppen et al., 2005), and knockdown of CTNNB1 had been shown to prevent tumorigenesis in this model (Delgado et al., 2015). In healthy livers, a downstream target of CTNNB1, i.e. glutamine synthetase (GS), is expressed around the central vein where there is a high concentration of WNT activity. Staining for GS is used as a marker for WNT activity. CTNNB1 tumors show ectopic GS expression that is outside of the central vein region. Because we saw an increase in HCC incidence in the siCtnnb1-treated group despite successful *Ctnnb1* knockdown, we evaluated expression of GS in DEN/PB livers. We expected to see widespread GS-positive tumors in the PBS mice, suggesting CTNNB1 activation. However, most tumors were GS-negative (Fig. 3F), suggesting that most of the tumors in this model were not driven by CTNNB1 activation. Consistently, siCtnnb1 was also ineffective at preventing these HCCs. In livers from the DEN/PB mice, CTNNB1, SMYD3 and CDK1 protein levels were reduced by 88%, 47% and 34%, respectively, while levels of ANLN remained unchanged (Fig. S3A,B). All changes in protein levels were significant when compared to those in PBS-treated mice, except for ANLN.

### Testing siRNA chemoprevention in a MASH HCC model

**Fig. 4.**
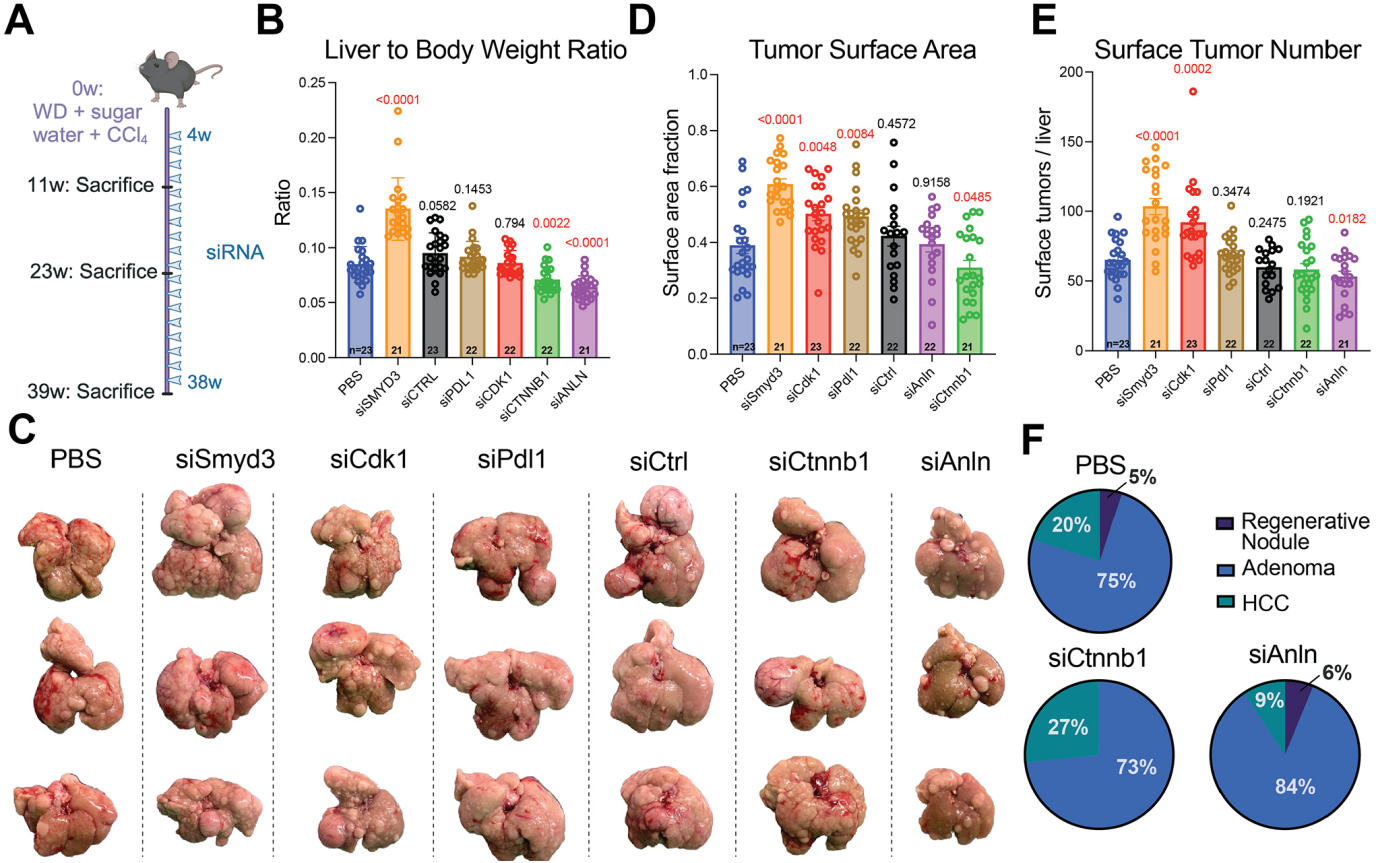
**siAnln decreased tumor burden in MASH mice.** (A) Schematic overview of the MASH model. At 8 weeks of age, male C57BL/6J mice were placed on WD and high-sugar water and began receiving weekly injections of CCl_4_ at the start timepoint of 0 weeks (0w). At 12 weeks of age, siRNA injections started (4w). Mice were treated with siRNA every other week and sacrificed at three different time points, i.e. 11, 23 and 39 weeks after WD initiation. (B) Liver-to-body-weight ratios at the 39-week time point on a MASH diet. (C) Representative images of livers from MASH mice at the 39-week time point. (D) Surface nodule area as measured using QuPath at the 39-week time point. (E) Surface tumor numbers at the 39-week time point. (F) Histological scoring of nodules and cancers at the 39-week time point. For panels B, D and E, the *P*-values with respect to the PBS group are shown above each group.

In the above models, there was no underlying liver disease that matched a relevant etiology of liver disease in human patients. Thus, we used a model of liver carcinogenesis caused by MASLD. A subset of MASLD patients develop MASH, an inflammatory, fibrotic disease that can lead to cirrhosis and HCC (Anstee et al., 2019). Mice with MASH (vs models using mutagens or oncogene overexpression) potentially mirror humans with chronic liver disease because fewer HCCs arise, and those that do arise develop with a longer latency. We used a dietary MASH model involving Western diet (WD) and high-sugar water in addition to CCl_4_, a hepatotoxin that causes liver injury (Tsuchida et al., 2018). This model mimics several aspects of fatty liver disease progression in humans, including histological abnormalities and changes in gene expression. Male C57BL/6J mice were started on a WD plus high-glucose/fructose water, and weekly low doses of CCl_4_ at 8 weeks of age. After 4 weeks on this regimen, when steatosis and inflammation – but not HCC  – were expected, subcutaneous siRNA injections were initiated and given every 14 days at a dose of 5 mg/kg of mouse (Fig. 4A). Mice were sacrificed at several time points in this model.

As expected, mice continuously gained body and liver weights throughout the study, but these changes were not uniform across groups. Discordant alterations in body vs liver weights were reflected in the liver-to-body-weight ratios. siCtnnb1 mice had decreased liver-to-body-weight ratios at 11 (Fig. S4A) and 39 weeks (Fig. 4B) due to increased body weights (Fig. S4B). siSmyd3 mice had increased liver-to-body-weight ratios at 11 (Fig. S4A) and 39 (Fig. 4B) weeks due to increased liver weights (Fig. S4C). siCtrl mice had an increased ratio at 11 weeks (Fig. S4A), and an increase that trended towards significance at 39 weeks (Fig. 4B), due to increased liver weights (Fig. S4C). At 39 weeks, siAnln mice had a decreased liver-to-body-weight ratio (Fig. 4B) due to a relative decrease in liver weight (Fig. S4C).

At the 39-week endpoint of the study, all mice had gross liver nodules (Fig. 4C). We analyzed these liver nodules in two ways in order to capture overall nodule burden in addition to nodule number. We first evaluated burden by assessing the proportion of the liver surface covered by nodules. This showed that the group of siCtnnb1-injected mice was the only one showing less liver surface area covered by nodules. Livers of siCtnnb1-treated mice showed 0.31±0.13 (mean±s.d.) coverage by nodules, compared to 0.39±0.14 or 0.39±0.12 in PBS- or siAnln-injected groups, respectively (Fig. 4D). Interestingly, some groups had an increase in nodule burden. The livers of siSmyd3-, siCdk1- and siPdl1-treated mice (0.61±0.09, 0.50±0.11 and 0.49±0.11, respectively) were covered by nodules (Fig. 4D). To evaluate nodule number, we counted the absolute number of liver nodules on the front and back of livers. This showed that siAnln-treated mice was the only group that had decreased nodule number (Fig. 4E). There were 53.1±17.3 nodules/liver in the livers of siAnln-treated mice compared to 65.1±14.4 nodules/liver in the livers of PBS-treated mice. Some groups had increased nodule numbers. There were 103.4±26.6 nodules/liver in livers of siSmyd3-treated mice and 91.9±27.5 nodules/liver in those of siCdk1-treated mice. Because these nodules could either be benign (i.e. regenerative or cirrhotic nodules) or malignant (HCC, cholangiocarcinoma, adenoma), we performed histopathological analysis in the PBS, siCtnnb1 and siAnln groups. For this, a single hematoxylin and eosin (H&E)-stained section of the largest lobe of each liver was analyzed. Although there were many nodules, only a small proportion were HCCs. Of the 20 nodules seen in the PBS group, 5% were regenerative nodules, 75% adenomas and 20% HCCs (Fig. 4F). Of the 15 nodules seen in the siCtnnb1-treated group, 73% were adenomas and 27% HCCs (Fig. 4F). Of the 32 nodules seen in the siAnln-treated group, 6% were regenerative nodules, 84% adenomas and 9% HCCs (Fig. 4F). Ultimately, this showed us that only a fraction of the nodules are true HCCs, but that the proportion of HCCs was similar between treatment groups. Thus, overall nodule quantification represented a surrogate for HCC numbers. We conclude that suppression of some gene targets could lead to increased cirrhosis and tumorigenesis, and that both siCtnnb1 and siAnln could confer modest protection against tumorigenesis in this MASH model. In the livers of these mice, PD-L1, ANLN, SMYD3, CDK1 and CTNNB1 protein levels were reduced by 50%, 47%, 23%, 9% and 4%, respectively (Fig. S4A, S4B). All changes in protein levels were significant when compared those in PBS-injected mice, except for CTNNB1.

### ANLN GalNAc siRNA treatment improved steatosis in a MASH HCC model

**Fig. 5.**
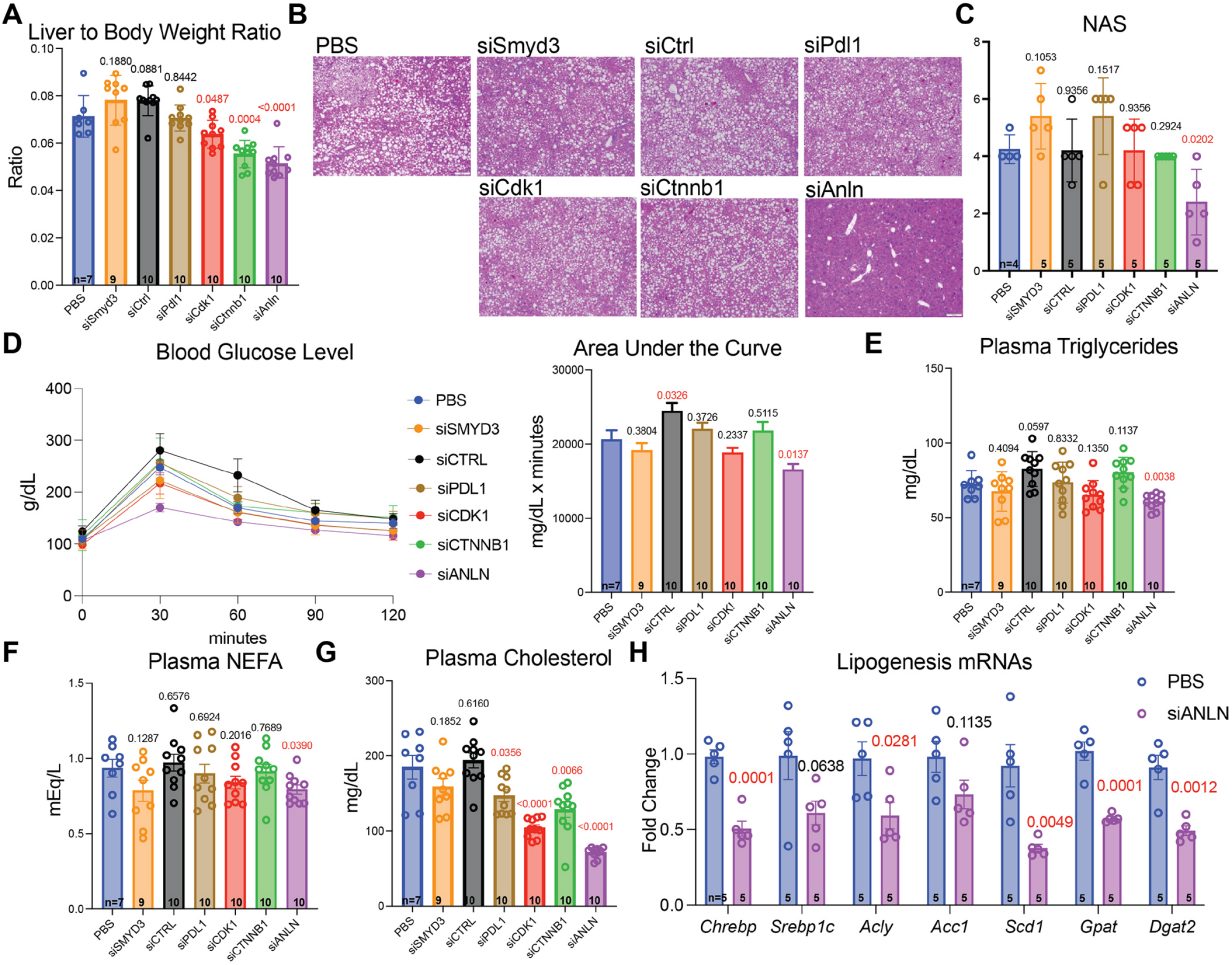
**siAnln treatment improved steatosis in the MASH HCC model.** (A) Liver-to-body-weight ratios after 23 weeks on WD and CCl_4_. (B) H&E after 23 weeks on WD and CCl_4_. Scale bars: 500 μm. (C) Non-alcoholic fatty liver disease (NAFLD) activity score (NAS) after 23 weeks on WD and CCl_4_. (D) Glucose tolerance test after 23 weeks on WD and CCl_4_. Area under the curve is shown to the right. (E-G) Plasma tests after 23 weeks on WD and CCl_4_. NEFA, non-esterified fatty acids. mEq/L, milliequivalents per liter. (H) qPCR for mRNAs associated with *de novo* lipogenesis. For panels A, C−H, the *P*-values with respect to the PBS group are shown above each group.

While preventing malignant transformation was the primary goal of our strategy, a concurrent amelioration of MASH would also be clinically beneficial. Hepatic steatosis, inflammation and fibrosis are histologic features that characterize MASH, which can ultimately lead to HCC. To better understand siRNA effects on MASH, we analyzed livers at the 23-week time point, which preceded most tumorigenesis. siCdk1, siCtnnb1 and siAnln groups had reduced liver-to-body-weight ratios (Fig. 5A), suggesting improvements in liver fat accumulation. Other than siAnln mice, all groups – including PBS controls – showed profound steatosis. The siAnln group exhibited a dramatic reduction in steatosis (Fig. 5B), which was in part responsible for the reduction in liver weights (Fig. S5A). The reduction in liver-to-body-weight ratio for siCtnnb1 mice was likely driven by the increase in body weight (Fig. S5B). Pathological scoring revealed that livers of siAnln-treated mice showed decreased levels of macrosteatosis (Fig. S5C) but no differences in microsteatosis (Fig. S5D) or hepatocyte hypertrophy (Fig. S5E). These three parameters together constitute the rodent non-alcoholic fatty liver disease (NAFLD) activity score (NAS), and only siAnln-treated mice had a decreased score (Fig. 5C). There were no significant differences in inflammation between any of the groups (Fig. S5F).

We sought to assess the underlying metabolic mechanisms responsible for differences in MASH. MAFLD is frequently driven by whole-body insulin resistance, which results in increases in adipocyte lipolysis, and subsequent increases in plasma triglycerides and non-esterified fatty acids (NEFAs). To measure insulin resistance, we performed glucose tolerance testing (GTT) on all groups. Only siAnln-treated mice had improved glucose tolerance, with reduced blood glucose levels at all time points of the GTT. siAnln-treated mice had no change in non-fasting insulin, suggesting that increased glucose tolerance was due to increased insulin sensitivity rather than increased insulin production (Fig. 5D). Consistent with improvements in insulin sensitivity, we found that siAnln mice had lower levels of plasma lipids, including triglycerides, NEFAs and cholesterol (Fig. 5E-G). This also suggested no increase in triglyceride or cholesterol export in livers of siAnln-treated mice. Injections of siPdl1, siCdk1 or siCtnnb1 also decreased plasma cholesterol but, due to a lack of other metabolic changes, we decided to focus solely on siAnln in subsequent studies. In addition to insulin resistance, another common contributor to MASH is increased dietary intake of fat, but we did not detect a gross change in food consumption or body weight (Fig. S5G). Finally, qPCR revealed that mRNA levels of multiple *de novo* lipogenesis genes, such as *Chrebp* (officially known as *Mlxipl*), *Srebp-1c* (officially known as *Srebf1*), *Acly*, *Dgat2*, *Gpat* (officially known as *Gpam*) and *Scd1* were decreased in in livers of siAnln-treated mice (Fig. 5H). In conclusion, siAnln-treated mice had less fatty liver due to increased insulin sensitivity, decreased circulating lipids and decreased *de novo* lipogenesis gene expression.

### Genetic ANLN deletion is effective for MASH and HCC

**Fig. 6.**
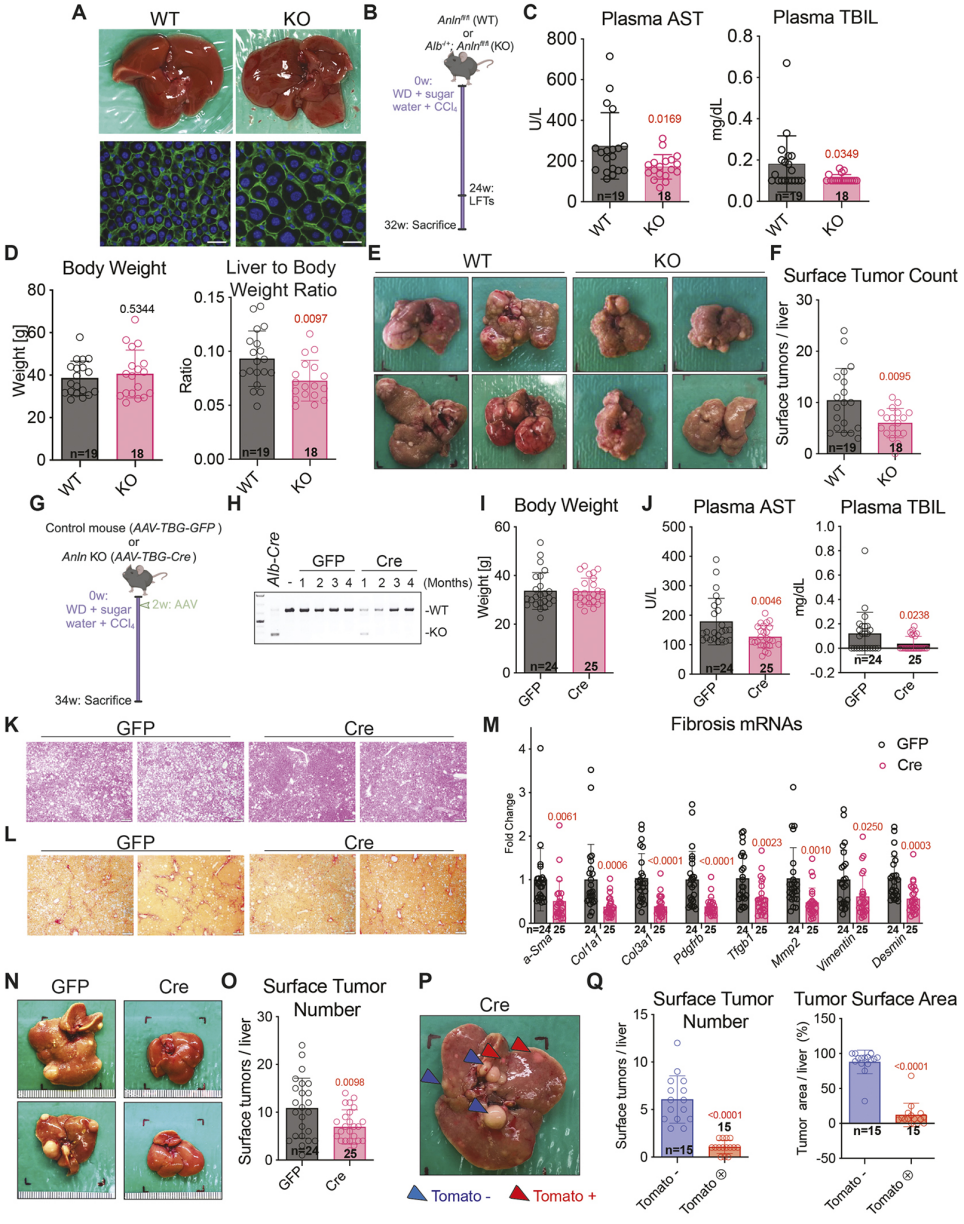
**Genetic deletion of *Anln* reduced steatosis, fibrosis, and HCC development.** (A) Top images: Comparison of whole liver obtained from wild-type (WT) and *Anln*-deficient (KO) mice shows no obvious differences in size or development. Bottom images: Immunofluorescence images of liver tissue showing increased polyploidy and cell size in liver tissue of the KO mouse. Immunostaining was for CTNNB1 protein (green); nuclei were stained with DAPI (blue). Scale bars: 15 µm. (B) Schematic of the MASH model applied to liver-specific *Anln* KO (*Alb*^−/+^*; Anln^fl/fl^*) and WT control mice (*Anln^fl/fl^*). At 8 weeks of age, mice were given a Western diet (WD) and high-sugar water, and began receiving weekly injections of CCl_4_, i.e. timepoint 0 weeks (0w). Plasma was taken from mice after 24 weeks (24w) and mice were sacrificed after 32 weeks (32w). (C) Plasma tests after 24 weeks on the MASH diet. Plasma AST (left) and total bilirubin (right) levels in liver-specific KO and WT mice. (D) Body weight (left) and liver-to-body-weight ratio (right) of KO vs WT mice. (E) Representative gross liver images of WT and KO mice. (F) Liver surface tumor numbers in WT and KO mice. (G) Schematic of the MASH model applied to *Anln^fl/fl^* mice given *AAV-TBG-GFP* or *AAV-TBG-Cre*. At 6 weeks of age, mice were given WD and high-sugar water, and began receiving weekly injections of CCl_4_ (0w). After two weeks (2w), mice were injected with AAV. Mice were sacrificed after 34 weeks (34w). (H) PCR of liver genomic DNA from *Anln^fl/fl^; LSL-tdTomato^+/+^* mice after *AAV-TBG-GFP* or *AAV-TBG-Cre* administration (WT amplicon: 700 bp; mutant amplicon: 300 bp). (I) Body weight levels in *AAV-TBG-GFP*- or *AAV-TBG-Cre*-treated mice. (J) Plasma AST (left) and total bilirubin (TBIL) (right) levels in *AAV-TBG-GFP*- or *AAV-TBG-Cre*-treated mice. (K) H&E from *AAV-TBG-GFP* or *AAV-TBG-Cre* treated mice after 34 weeks on the MASH diet. Scale bars: 500 μm. (L) Sirius Red staining on livers after 34 weeks on the MASH diet. Scale bars: 500 μm. (M) qRT-PCR analysis performed on livers after 34 weeks on the MASH diet. (N) Representative gross liver images. (O) Number of surface tumors/liver of *AAV-TBG-Cre*- or *AAV-TBG-GFP*- treated mice. (P) Representative gross liver image of *Anln^fl/fl^; LSL-tdTomato^+/+^* mice after *AAV-TBG-Cre* and 34 weeks on MASH diet. Blue arrows point to tdTomato-negative (Tomato –) tumors; red arrows point to tdTomato-positive (Tomato +) tumors. (Q) Number of surface tumors/liver (left) and tumor area/liver (right) of tdTomato-positive vs tdTomato-negative (Tomato + vs Tomato −, respectively) tumors in each liver. *P*-values in panels C, D and F are shown with respect to the WT group; *P*-values in panels I, J, M and O are shown with respect to the GFP group; *P*-value in panel Q is shown with respect to the tdTomato-negative group.

Given that the siAnln caused variable knockdown, but was the most effective at HCC prevention, we sought to further determine if the MASH and HCC effects of siAnln were on-target and non-toxic. We generated *Anln* floxed mice that permitted conditional genetic deletion by using two Cre-based methods. First, we used albumin-*Cre*, which causes complete deletion of genes in all parenchymal cells of the liver (hepatocytes and bile duct epithelial cells) starting during embryogenesis. A disadvantage of albumin*-Cre* is that gene deletion begins early in embryogenesis, leading to much higher levels of polyploidization than if ANLN deletion is induced in adults. Despite this, *Anln*-deficient mice were born with the expected Mendelian ratios and livers developed normally without changes in organ size or function (Fig. 6A). Even though this extreme level of polyploidization is unlikely to be achieved in humans, the viability of these mice supports the safety and tolerability of complete ANLN elimination from hepatocytes and biliary epithelial cells. Next, wild-type (WT) control mice (*Anln*^*fl/fl*^) and Anln KO mice (*Alb*^*−/+*^*; Anln*^*fl/fl*^) cohorts were started on the chronic MASH to HCC model using WD and high-sugar water, as well as weekly CCl_4_ injections for 32 weeks (Fig. 6B). To test if liver-specific *Anln* deletion prevents liver damage from MASH, we obtained plasma liver function tests after 24 weeks. *Anln* KO reduced levels of plasma aspartate aminotransferase (AST) and total bilirubin (TBIL), used as markers of liver injury and inflammation, respectively (Fig. 6C). This suggests that *Anln* deletion helped to protect against liver injury during MASH. After 32 weeks, liver-specific *Anln* deletion did not affect body weight but significantly decreased the liver-to-body-weight ratio (Fig. 6D). This was partially due to the decrease in number and size of liver surface tumors (Fig. 6E,F). These genetic data indicate that high-potency embryonic deletion of *Anln* prevents MASH-induced tumor formation.

To mimic the siRNA mediated suppression of ANLN only in adult hepatocytes, we used an adeno-associated virus (AAV) that infects and expresses Cre recombinase only in hepatocytes (hereafter called *AAV-TBG-Cre*). This genetic approach mimics GlaNAC-siRNAs, which only deliver to hepatocytes. In order to lineage-trace the origin of HCC formation, we also introduced a Cre-inducible tdTomato reporter and generated *Anln^flox/flox^*; *LSL-tdTomato^+/+^* mice. We then repeated the chronic MASH to HCC model with this *AAV-TBG-Cre*-induced *Anln* KO model. Mice were initiated on WD, high-sugar water and weekly CCl_4_ injections at 6 weeks of age, then injected with AAV at 8 weeks of age. *Anln^fl/fl^* mice were randomly divided and subjected to *AAV-TBG-GFP* or *AAV-TBG-Cre* injection (Fig. 6G). *Anln* deletion was 90% effective at the DNA level 1 month after *AAV-TBG-Cre* injection (Fig. 6H). Over time, the fraction of undeleted ANLN alleles increased, potentially due to repopulation of the liver by *ANLN* heterozygous or WT clones (Fig. 6H). A second *AAV-TBG-Cre* dose, given 3 months after the first dose (month 4), was unsuccessful at increasing the fraction of *Anln* deleted cells (Fig. 6H).

After *Anln* deletion, no differences regarding body weight or death were seen (Fig. 6I). We also found that levels of blood marker proteins of liver injury and inflammation (i.e. AST and TBIL, respectively) improved at multiple time points during the MASH modeling. Plasma AST and TBIL levels were significantly lower in *AAV-TBG-Cre*-treated mice (Fig. 6J). Similar to mice injected with siRNA, steatosis was significantly reduced in livers of *Anln* KO mice after 12 weeks of the MASH regimen (Fig. 6K). At the final time point (34 weeks), there was reduced fibrosis as measured by Sirius Red staining and reduced mRNA expression of multiple fibrosis-related genes, i.e. *α-Sma*, *Col1a1*, *Col3a1*, *Pdgrfb*, *Tgfb1*, *Mump2*, vimentin and desmin (Fig. 6L,M). These data show that inhibition of *Anln* leads to the suppression of steatosis and fibrosis, corroborating the on-target activities of siAnln.

Despite the sub-optimal deletion of *Anln*, liver-specific *Anln* KO mice developed significantly fewer and smaller tumors by the 32-week time point (Fig. 6N,O). Because *Anln* was not completely deleted beyond the 4-week time point, we asked if tumors that did arise originated from non-deleted escaper clones. By quantifying tdTomato-positive tumors, we found that a majority of the tumors that arose from KO livers were tdTomato negative (Fig. 6P), suggesting that these tumors arose from non-deleted wild-type clones. Furthermore, the tdTomato-positive clones were much smaller than the negative clones, again suggesting that when successful, suppression of ANLN prevented tumor development (Fig. 6Q). Repopulation by ANLN-expressing clones in these models does not necessarily imply a regenerative defect in ANLN-negative hepatocytes. Instead, the expansion of ANLN-positive cells likely reflects a selective advantage under oncogenic or stress conditions. These rigorous reference standard genetic deletion results confirm the *in vivo* siRNA findings and support the potential safety of ANLN inhibition.

## DISCUSSION

Cancer chemoprevention, specifically for HCC, represents a large unmet clinical need and requirements for an ideal chemopreventive agent – safety and efficacy – have yet to be met. Here, we demonstrated that GalNAc-siRNA molecules are viable options for HCC chemoprevention in a variety of mouse models. In addition, we also identified a gene target, *ANLN*, that was most effective at preventing tumor formation. Genetic studies further supported the protective effect of ANLN knockdown in cancer formation.

When evaluating preclinical models of HCC formation and prevention it is important to balance clinical relevance and experimental feasibility. We attempted to account for the heterogeneity of human HCC by testing chemoprevention in multiple models of HCC. Successful chemoprevention will probably require the prevention of an array of HCC subtypes driven by different etiologies and driver oncogenes. We used multiple cancer models to rigorously test siRNA candidates and to capture important factors in HCC development, i.e. oncogenic driver genes; liver injury; and pre-existing diseases, such as MASH. HDT models are based on common oncogenic driver genes that cause rapid progression and robust tumor formation. While these models can provide insight into specific genetic mechanisms of HCC, they are aggressive and, thus, often differ from the slower development of many human HCCs. The DEN/PB model gives rise to tumors resulting from hepatocyte mutations and injury, which captures a subset of the genetic factors and injuries contributing to HCC. However, DEN and PB are not carcinogens that humans are routinely exposed to and, thus, fail to replicate some aspects of the genetic basis of human HCC. The MASH model we used is the most clinically relevant and has been shown to closely resemble human disease in both histological and transcriptomic analysis (Tsuchida et al., 2018). This model allowed us to test chemoprevention in the context of metabolic dysfunction and chronic liver injury. Even though this model yielded a low number of HCCs, it demonstrated that siRNAs can influence underlying etiologies that contribute to the formation of HCCs, such as MASH.

siAnln was effective at preventing and decreasing tumor formation across multiple, independent HCC models. For some of genes, such as *Ctnnb1* and *Cdk1*, targeting them by using siRNA was effective in only some of the models. Our current siAnln findings reinforce the findings from previous work (Lin et al., 2020; Zhang et al., 2018a,b). Knockdown of *Anln* leads to cytokinesis failure and, in turn, to a polyploid state within hepatocytes; and, while we achieved only ∼50% mRNA knockdown 3 days after injection, this level of suppression aligns with previous findings showing that even partial ANLN inhibition can have significant effects (Zhang et al., 2018b). Differences in knockdown efficiency probably reflect model-specific variation in oncogenic drivers, inflammation and hepatocyte turnover. To account for this variability, we used multiple tumor assays to support the robustness and context-independence of ANLN as a target for chemoprevention. Additionally, genetic studies have shown that *Anln^fl/+^* mice, which carry a heterozygous deletion of *Anln*, exhibit markedly reduced tumor development (Chen et al., 2022). Together, these findings suggest that even modest reductions in ANLN levels can disrupt tumorigenesis. Hepatocytes at baseline are polyploid, and ploidy changes follow major liver injury (Lin et al., 2020) or liver resection (Tamura et al., 1992). Prior work has demonstrated that polyploidy protects hepatocytes from malignant transformation (Zhang et al., 2018b). In addition to cancer protection, *Anln* knockdown has been shown to protect against liver injury without altering the regenerative capacity of the liver (Lin et al., 2020). This work here demonstrated that pharmacological knockdown of *Anln* by using GalNAc-siRNAs replicates the findings from previous work, inducing a natural state of hepatocytes that confers cancer protection.

While the use of GalNAc-siRNAs for chemoprevention is a new concept, siRNAs are a clinically approved mode of drug delivery for existing liver diseases. This allows us to take advantage of the hepatocyte-specific targeting by the GalNAc ligand and exploits the safety profile of these drugs in a novel way. Since patients who would receive HCC chemoprevention generally present with an underlying liver disease, the limited hepatic and extrahepatic toxicity associated with this class of liver-targeting molecules would be highly desirable. A key component of translating these findings into humans will be to determine the optimal dosing regimen in order to achieve maximum efficacy and minimal toxicity. Based on the preclinical evidence generated here, GalNAc-siRNAs have the potential to safely and effectively prevent liver cancer in patients with advanced liver disease.

## MATERIALS AND METHODS

### Mouse models of liver disease and cancer

#### Anln^f/f^ mice

LoxP sites were inserted around exon 2 using CRISPR injections.

#### tdTomato-positive mice

*Anln*^*fl/fl*^ and *tdTomato*^*fl/fl*^ were crossed to generate tdTomato-positive *Anln*^*fl/fl*^ mice mice. Mice received 1x10^11^ genome copies of AAV-CRE/AAV-GFP at 8 weeks of age. WD and low dose CCL4 once a week started at 6 weeks of age and lasted for 8 months. Liver and plasma were taken at the end points for functional analysis.

All mice were handled in accordance with the guidelines of the Institutional Animal Care and Use Committee at The University of Texas Southwestern Medical Center (UT Southwestern). All experiments began when mice were 8 weeks of age, except for the DEN/PB model which started when mice were 2 weeks of age. Each week, mice were weighed and examined for any physical injuries and signs of disease. Once a mouse reached a body condition score of 1 out of 5 (with 1= emaciated, 2= underconditioned, 3= well-conditioned, 3= overconditioned, 5= obese; see Ullman-Culleré and Foltz, 1999), the mouse was euthanized. When mice were sacrificed, harvested livers were photographed (front and back) and either placed into 10% formalin for histological analysis or snap frozen for other analysis. If relevant, cardiac blood was collected for plasma and metabolic tests, and sent to the Metabolic Phenotyping Core at UT Southwestern. To be included in the *CTNNB1/MYC* model Kaplan–Meier curve, mice had to receive at least three doses of siRNA. Mice in other models were excluded from analysis if they were sacrificed prior to the designated end date. Only male mice were used.

### Plasma measurements

Blood was collected from the heart immediately after sacrificing the mouse and centrifuged at 2000 ***g*** for 15 min at 4°C in heparinized Fisherbrand™ Microhematocrit Capillary Tubes (Thermo Fisher Scientific, #22-362566). After centrifugation, the supernatant (plasma) was transfered to a clean 1.5 ml tube and sent to the UT Southwestern Metabolic Phenotyping Core for measurement of aspartate aminotransferase (AST) and total bilirubin (TBIL), usesd as markers of liver injury and inflammation, respectively.

#### siRNAs

Chemically modified and functionalized siRNAs for this study were synthesized and characterized as reported previously (Foster et al., 2018) and LNP-formulated siRNAs were prepared as described elsewhere (Maier et al., 2013). siRNAs targeting *Cdk1*, *Pdl1*, *Ctnnb1*, *Anln*, *Smyd3* or luciferase were resuspended in filtered PBS, aliquoted and stored at −20°C until use. Additional aliquots of PBS were made as a PBS-only vehicle control.

#### Hydrodynamic transfection (HDT) models

c-myc-PT3EF1a (Addgene plasmid #92046), pT3-EF1aH N90-CTNNB1 (Addgene plasmid #86499) and SB100 were gifts from Xin Chen (UCSF, San Fransisco, CA, USA). T/Caggs-NRASV12 (Addgene plasmid #20205) and pT2/shp53/GFP4 (Addgene plasmid #20208) were gifts from John Ohlfest (University of Minnesota, MN, USA). Plasmids for HDT injection were diluted in sterile saline [10 μg of each oncogenic plasmid and 2 μg of the Sleeping Beauty Transposase SB100 (Tward et al., 2007) per 2 ml of saline] prior to tail vein injection in male C57BL/6J mice. If the body weight of the mouse was <20 g or >23 g, the injection volume was 10% of its body weight. If the body weight of the mouse was 20-23 g, it received a 2 ml injection. Mice were injected ∼8 weeks of age. siRNA doses were given subcutaneously at a dose of 5 mg/kg every 14 days starting 3 days after HDT unless otherwise specified.

#### DEN/PB model

N-Nitrosodiethylamine (diethylnitrosamine, DEN) (Sigma-Aldrich, #N0756) was diluted in sterilized PBS and administered via intraperitoneal injection at a dose of 25 mg/kg when male C3H mice were 14 days of age. At 6 weeks of age, phenobarbital (PB) (Sigma Aldrich, #P5178) was added to their drinking water at a final concentration of 0.05% w/v. siRNA (at 5 mg/kg) doses were given subcutaneously every 14 days starting at 12 weeks of age.

#### MASLD model

The metabolic dysfunction-associated steatotic liver disease (MASLD) model was based on a previous publication (Tsuchida et al., 2018). Male C57BL/6J mice were fed a Western diet (WD) of 42% Kcal from fat with sucrose and 1.25% cholesterol (Inotiv, TD.120528) and high-sugar water (23.1 g/l D-fructose, Sigma-Aldrich, #F0127 and 18.9 g/l D-glucose, Sigma-Aldrich, #G8270) starting at 8 weeks of age. Mice received weekly intraperitoneal injections of CCl_4_ (Sigma Aldrich, #289116) in corn oil (Sigma Aldrich, #C8267) or a corn oil-only vehicle injection at a dose of 0.32 μg CCl_4_/g of mouse body weight or the equivalent volume of corn oil at 8 weeks of age. siRNA doses (5 mg/kg) were given subcutaneously every 14 days starting at 12 weeks of age. Glucose tolerance tests were performed after 11, 24 and 39 weeks on WD, 5 days after the most-recent CCl_4_ injection. Mice were fasted overnight for 16 h before analysis of baseline blood glucose level (*t*=0). Blood was taken from a small incision on a lateral tail vein. A 10% glucose solution in saline was intraperitoneally injected at a dose of 10 μl/g of body weight. Subsequent blood glucose levels were measured at 30, 60, 90 and 120 min after glucose injections. All glucose readings were taken using a Life-scan One Touch glucometer.

#### *Alb-Cre* model

*Alb-Cre^−/+^* mice were crossed to *Anln^fl/fl^* mice to generate liver specific *Anln* KO mice (*Alb-Cre^−/+^; Anln^fl/fl^*) and control mice (*Anln^fl/fl^*). At 8 weeks of age, both groups were put on the MASLD diet described above. Tissues for functional analysis were collected 32 weeks thereafter.

#### *AAV-TBG-Cre* model

*Anln^fl/fl^* mice were randomly assigned into two different groups, i.e. *AAV-TBG-Cre* and *AAV-TBG-GFP*. MASLD diet as described above was started at 6 weeks of age. Retro-orbital injection of AAV virus at 1×10^11^ genome copies/mouse was given at 8 weeks. Tissues for functional analysis were collected 32 weeks thereafter.

### Tumor analysis and quantification

For tumor analysis, frontal and rear images of each liver were taken. Tumors on each side were manually counted and added together to calculate the total number of surface tumors/liver. Tumor surface area was calculated in QuPath.

### Liver histology and immunostaining

Formalin-fixed and paraffin-embedded slides were stained with hematoxylin and eosin (H&E) or Picro Sirius Red (Abcam, #150681). The non-alcoholic fatty liver disease (NAFLD) activity score (NAS) was determined by a pathologist who scored the slides according to previously published rodent guidelines (Liang et al., 2014). The scoring carried out by a pathologist unaware of the identity of siRNA groups. For immunofluorescence staining, slides were deparaffinized and dehydrated prior to blocking in 10% goat serum in PBST. Slides were stained using the following antibodies: anti-glutamine synthetase (Abcam, #ab73593, 1:1000), Alexa Fluor 594 donkey anti-rabbit IgG (Life technologies, #A21207, 1:1000), HNF4a (Invitrogen, #417800, 1:500) and anti-Ki67 (Abcam #ab15580, 1:500).

### Western blots

Snap-frozen livers were homogenized in T-PER Tissue Protein Extraction Reagent (Life Technologies, #78510) supplemented with protease and phosphatase inhibitors (#501905532 and #501905547, respectively; both Fisher Scientific). Protein levels were quantified using a Pierce BCA Protein Assay kit (Thermo Fisher Scientific, #23227). Samples were mixed with 6× Laemmli SDS Sample buffer (Boston BioProducts, BP-111R) and heated to 95°C for 10 min prior to loading on a 4-20% gradient Tris-Glycine SDS mini gel (Bio-Rad, #4561096). Per sample, 20 μg of protein was loaded. The following antibodies were used: anti-CDK1 (Abcam, #ab32094, 1:1000); anti-catenin B (BD, #610154, 1:1000); anti-Smyd3 (Abcam, #ab187149, 1:1000); anti-Anillin (Abcam, #ab154337, 1:500); anti-PD-L1 (Abcam, #ab213480, 10:1000); anti-beta Actin (Cell Signaling Technology, #4970, 1:5000); anti-vinculin (Cell Signaling Technology, #13901, 1:10000).

### RT-qPCR

Snap-frozen liver samples were homogenized in Trizell (Thermo Fisher Scientific, #15596026) prior to RNA extraction. cDNA was synthesized using an iScript cDNA synthesis kit (Bio-Rad, #1708891). Primers used for qPCR are shown Table S1.

### Statistical analysis

Statistical analysis was done using two-tailed *t*-test and data were compared to the PBS group. Variation is represented as mean± standard deviation (±s.d.). Mice were randomized prior to receiving the first dose of siRNA based on average cage weight. There were no significant differences between any of the groups at the start of the experiment, and mice were not discarded once the study had been initiated. We used independent two-tailed *t*-tests to compare each treatment group to the PBS group. We did not correct for multiple hypothesis testing. While this can increase the chance of false positives, we interpret the results with this possibility in mind. Statistical significance is defined as *P*<0.05.

## Supplementary Material

10.1242/dmm.052370_sup1 Supplementary information
