# Supplementary material for: Chemoprevention of hepatocellular carcinoma using N-acetylgalactosamine-conjugated siRNAs
Source: Dis Model Mech. 2025 Sep 1;18(8):dmm052370. doi: 10.1242/dmm.052370 (PMC12452061; doi:10.1242/dmm.052370)
Supplement: Supplementary information [file dmm-18-052370-s1.pdf]

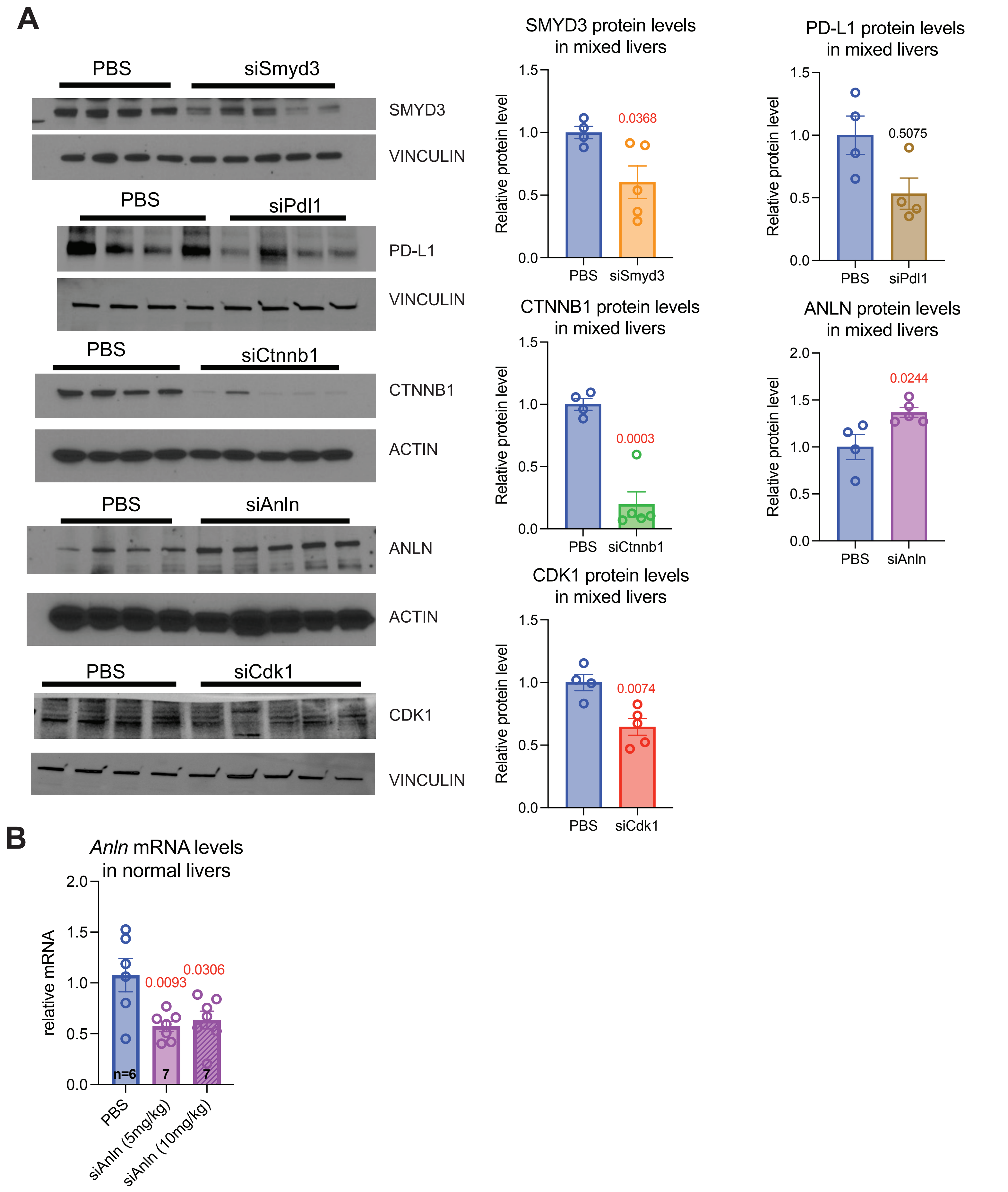

Fig. S1. Protein expression in liver tissues from the *CTNNB1/MYC* HCC model.
A. Western blots of liver tissues collected from the *CTNNB1/MYC* HDT model. The liver tissue contains a mixture of tumor and normal tissues. Quantification of Western blots to the right. B. qPCR for *Anln* mRNA in normal livers. C57BL/6J mice were injected with siANLN and sacrificed 3 days later. For A and B, the P-values with respect to the PBS group are shown above each group.

Disease Models & Mechanisms • Supplementary information

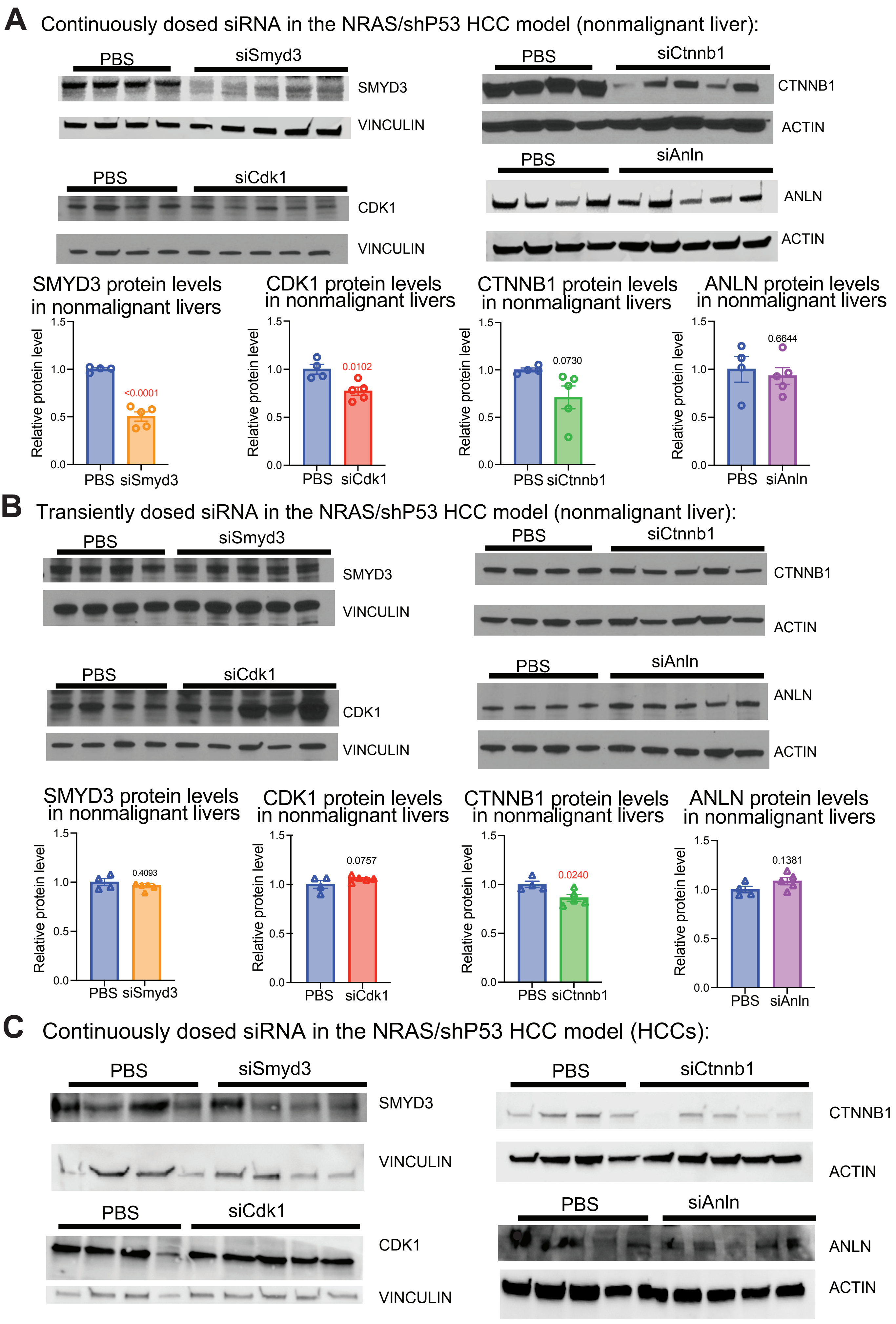

Fig. S2. Western blots of livers from the *NRAS^{G12V}/shp53* HCC model. A. Western blot of non-malignant liver tissue from mice treated with continuous siRNA dosing. Livers taken 7.5 weeks after HDT. Quantification shown below.

B. Western blot of non-malignant liver tissue from mice treated with transient siRNA dosing. Livers taken 7.5 weeks after HDT. Quantification shown below.

C. Western blot of HCCs taken from mice treated with continuous siRNA dosing.

For A and B, the P-values with respect to the PBS group are shown above each group.

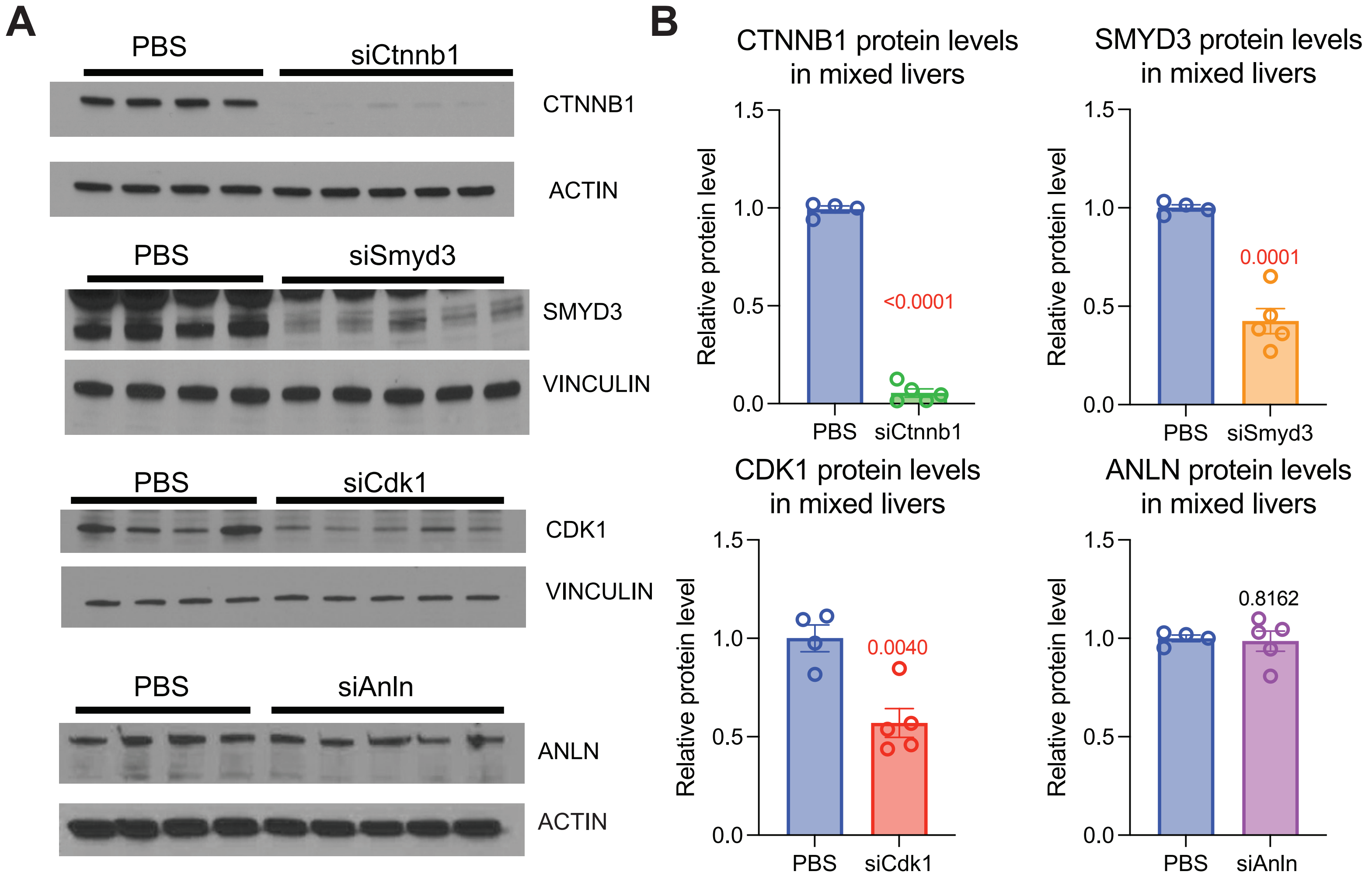

Fig. S3. Western blots of livers from the DEN/PB HCC model.

A. Western blot of livers taken from DEN/PB treated mice sacrificed at the end point. The liver tissue contains a mixture of tumor and normal tissue.

B. Quantification of Western blots. The P-values with respect to the PBS group are shown above each group.

Disease Models & Mechanisms • Supplementary information

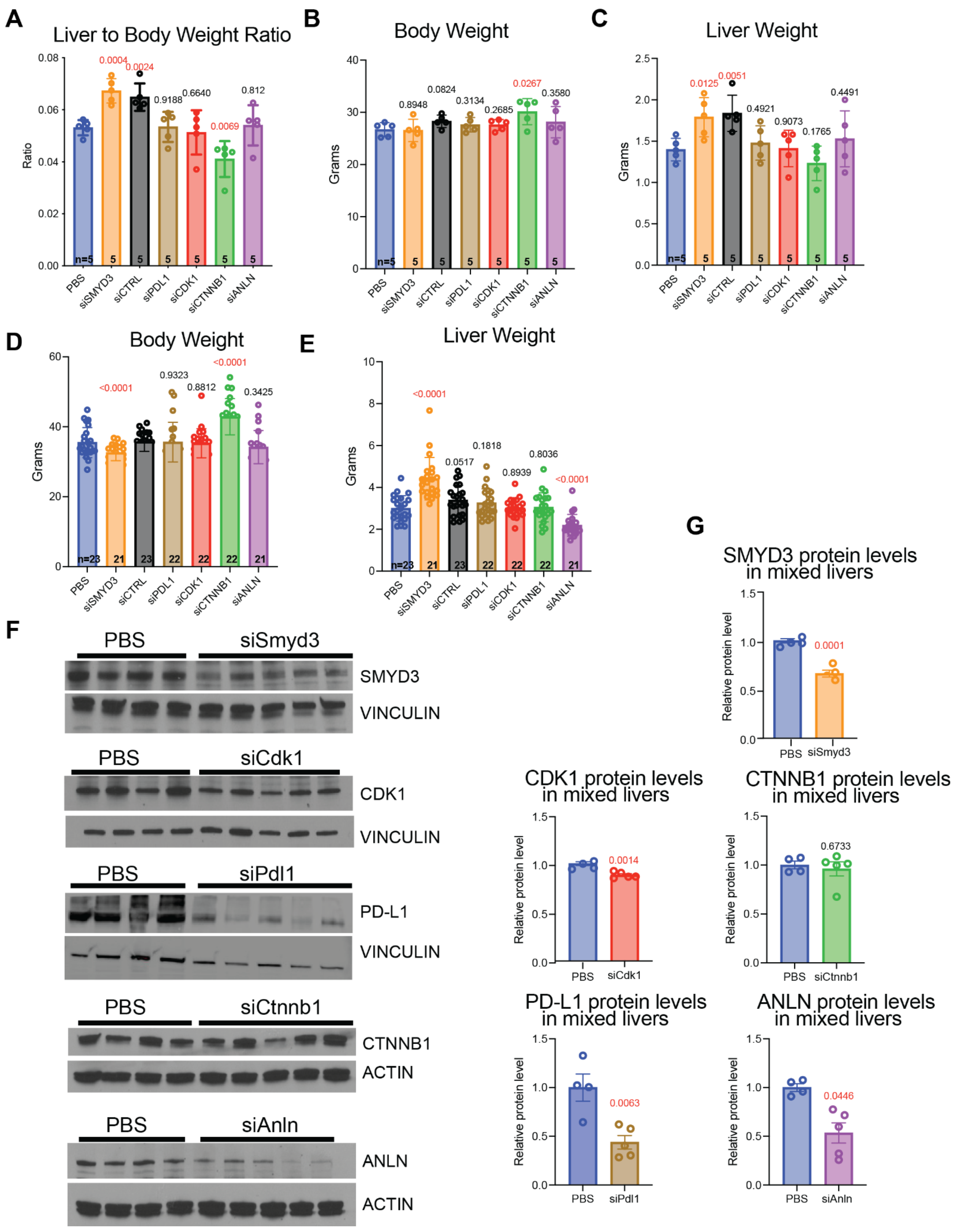

Fig. S4. Additional data collected from the MASH mice.

A-C. Body weight, liver weight, and liver to body weight ratio after 11 weeks on the MASH diet. D-E. Body weight and liver weight after 39 weeks on the MASH diet.

F. Western blots of MASH liver after 39 weeks on the MASH diet. The liver tissue contains a mixture of tumor and normal tissue.

G. Quantification of Western blots.lo

For several panels, the P-values with respect to the PBS group are shown above each group.

Disease Models & Mechanisms • Supplementary information

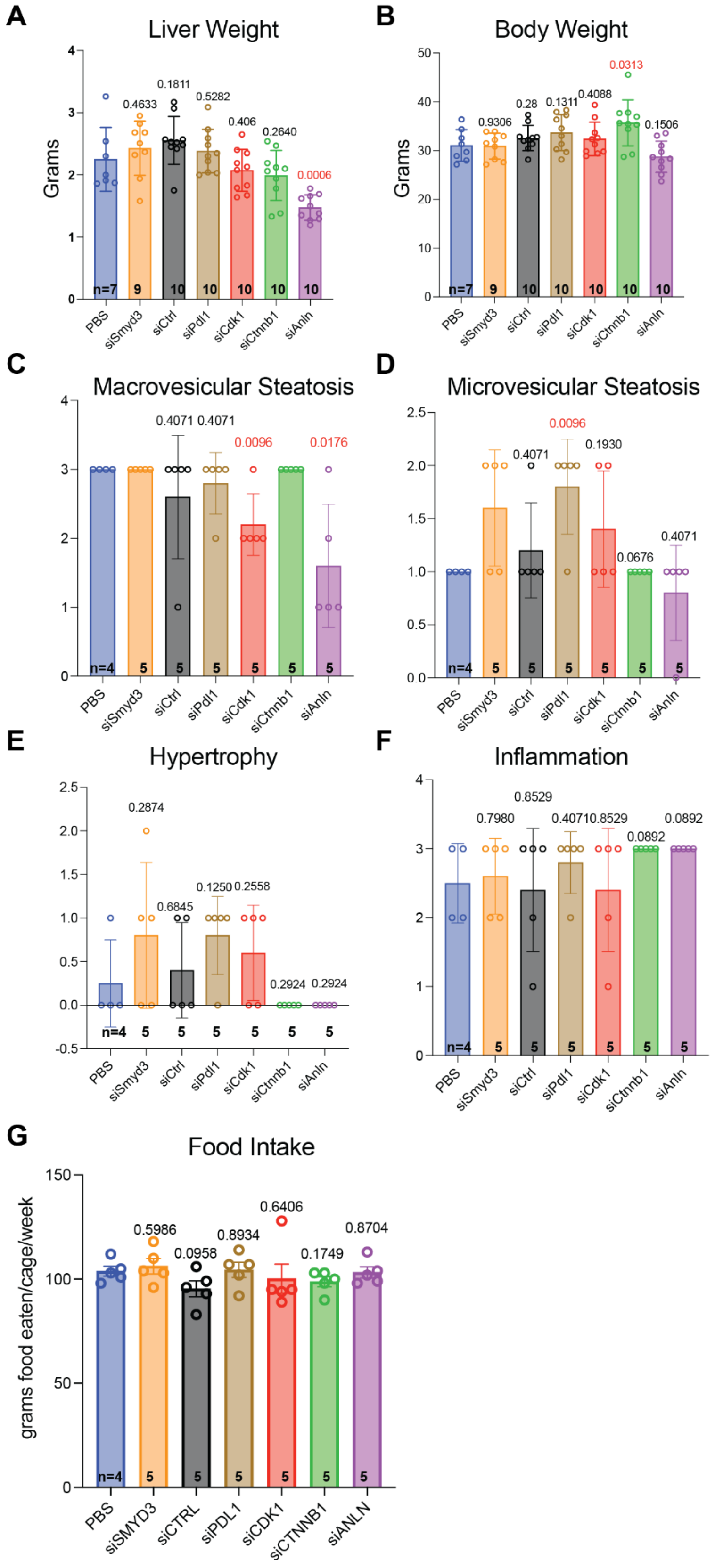

Fig. S5. Data collected at the 23 week MASH time point.

A. Liver weights after 23 weeks on the MASH diet.

B. Body weights after 23 weeks on the MASH diet.

C. Macrovesicular steatosis assessed by a pathologist.

D. Microvesicular steatosis assessed by a pathologist.

E. Hypertrophy.

F. Inflammation.

G. Food intake measured per cage over the course of a week.

For all panels, the P-values with respect to the PBS group are shown above each group.

Disease Models & Mechanisms • Supplementary information

Table S1. Primers used for qPCR.

Primer/gene name	Forward sequence (5′−3′)	Reverse sequence (5′−3′)
Chrebp	CTGGGGACCTAAACAGGAGC	GAAGCCACCCTATAGCTCCC
Srebp1c	TGCTCCAGCTCATCAACAAC	AGAGAGGAGGCCAGAGAAGC
Acly	TGTTGACATTGGAGCCCTC	GATACAGCCCTTGCTTCAG
Acc1	TGAGGAGGACCGCATTTATC	CATGGGATGGCAGTAAGGTC
Scd1	CCGGAGACCCTTAGATCGA	TAGCCTGTAAAAGATT TCTGCA AACC
Gpat	CAACACCATCCCCGACATC	GTGACCTTCGATTATGCGATCA
Dgat2	CTGTGCTCTACTTCACCTGGCT	CTGGATGGGAAAGTAGTCTCGG
α-Sma	GAGAAGCCCAGCCAGTCG	ATCTTTTCCATGTCGTCCCAGTTG
Col1a1	TTCTCCTGGCAAAGACGGACTCAA	AGGAAGCTGAAGTCATAACCGCCA
Col3a1	AACCTGGTTTCTTCTCACCCTTC	ACTCATAGGACTGACCAAGGTGG
Pdgrfb	TTCCAGGAGTGATACCAGCTT	AGGGGGCGTGATGACTAGG
Tgfb1	CTCCCGTGGCTTCTAGTGC	GCCTTAGTTTGGACAGGATCTG
Mump2	CAAGTTCCCCGGCGATGTC	TTCTGGTCAAGGTCACCTGTC
Vimentin	ACCGCTTTGCCAACTACAT	TTGTCCCGCTCCACCTC
Desmin	GTGGATGCAGCCACTCTAGC	TTAGCCGCGATGGTCTCATAC

Disease Models & Mechanisms • Supplementary information
